# FDI technology spillover and threshold effect of the technology gap: regional differences in the Chinese industrial sector

**DOI:** 10.1186/s40064-016-1962-6

**Published:** 2016-03-12

**Authors:** Hui Wang, Huifang Liu, Zhiyong Cao, Bowen Wang

**Affiliations:** School of Humanities and Economic Management, China University of Geosciences, No. 29 Xueyuan Road, Haidian District, Beijing, 100083 People’s Republic of China

**Keywords:** FDI technology spillover, Technology gap, Threshold effect, Chinese industrial sectors

## Abstract

This paper presents a new perspective that there is a double-threshold effect in terms of the technology gap existing in the foreign direct investment (FDI) technology spillover process in different regional Chinese industrial sectors. In this paper, a double-threshold regression model was established to examine the relation between the threshold effect of the technology gap and technology spillover. Based on the provincial panel data of Chinese industrial sectors from 2000 to 2011, the empirical results reveal that there are two threshold values, which are 1.254 and 2.163, in terms of the technology gap in the industrial sector in eastern China. There are also two threshold values in both the central and western industrial sector, which are 1.516, 2.694 and 1.635, 2.714, respectively. The technology spillover is a decreasing function of the technology gap in both the eastern and western industrial sectors, but a concave curve function of the technology gap is in the central industrial sectors. Furthermore, the FDI technology spillover has increased gradually in recent years. Based on the empirical results, suggestions were proposed to elucidate the introduction of the FDI and the improvement in the industrial added value in different regions of China.

## Background

Since the 1990s, a significant amount of foreign direct investment (FDI) has flowed into China and has grown rapidly. According to the World Investment Report (2015) released by the UN’s trade development organization, there was approximately $129 billion in FDI flowing into China in 2014, which was a record high. China has been the largest country in terms of FDI flows in the world. FDI could generate technology spillover to the host country through domestic enterprises’ acquisition of high technical knowledge from foreign-funded enterprises; the domestic enterprises compete with foreign-funded enterprises in the products and the human capital that flows between the domestic enterprises and foreign-funded enterprises. Furthermore, whether the technology spillover that occurred was positive depended on the absorptive capacity. The absorptive capacity includes the degree of the regional innovation, the education level, the financial market development, the economic development, the quantity of human capital, the technology gap level and so on. Additionally, the technology gap level, which was regarded as an important factor of absorptive capacity, was direct and effected the significance of technology spillover.

However, the eastern, central and western regions of China were difference in the location conditions, the reform and opening process and the marketization degree, so it is clear that the technology gap between the domestic and foreign industrial sectors was different among the three regions. Additionally, the threshold effect in terms of the technology gap existing in the FDI technology spillover process in different regional Chinese industrial sectors. To find the relation between the threshold effect of the technology gap and the FDI technology spillover in different regional Chinese industrial sectors, it is necessary for us to research it. The remainder of this paper is organized as follows: the following section is the literature review; third section introduces the “Model and data”; fourth section focuses on the “Empirical analysis”, fifth section ends with “Conclusions and policy recommendations”.

## Literature review

Evidence from the existing literature showed that the issue of FDI technology spillover has aroused scholar interest for many years. Caves (1974), Globerman (1979), Blomström (1986), Kokko (1994), Dimelis and Louri (2002), Javorcik and Spatareanu (2008), Suyanto and  Salim  (2013), Jude (2015) had conducted research on Canada and Australia, Canada, Mexico, Uruguayan, Portugal, Greece, Romania, Indonesia, and Romania respectively, and the positive spillovers were indeed founded. Moreover, the majority of Chinese researchers also gained strong support for FDI positive technology spillovers in China. He and Xu (1999) used the time-series data of the industrial sector from 1985 to 1996 and found that FDI had positive technology spillovers to the domestic industrial sector. Pan (2003) used the China’s industrial sectors panel data from 1995 to 2000 and found that the spillover effects of FDI on China’s industrial sectors were positive. By applying the province level panel data between 1993 and 1994, Xie (2006) found that FDI had a significant spillover to improve the technical efficiency. Kuo and Yang (2008) also found that FDI could generate spillover and had contribution to the regional economic growth of China. Yu (2011) also revealed that FDI had positive technology spillover to promote technological progress. Zhang et al. (2014) used the inter-provincial panel data of China from 1998 to 2012 and one-stage SFA method to study the FDI spillover effects and found that FDI could bring significant technology spillover effects for China. Yi et al. (2015) revealed that FDI had positive spillover on the domestic firms in different regions of China, but it did not equally.

Moreover, Cohen and Levinthal (1989) first noted that whether the technology spillover effect really occurred depended on the absorptive capacity. And Girma (2005) argued that there was a absorptive capacity threshold level which could decide whether the FDI technology spillover was positive or negative. By reviewing the related literature, there were many scholars who tested the above variables as the factor of absorptive capacity in their research; in particular, the technology gap had been tested by many scholars who found a threshold effect in terms of a technology gap in the technology spillover process. The threshold effect refers to when the technology gap passes the threshold value; the FDI technology spillover is significantly different than before. There were two types of views regarding the threshold effect of the technology gap in FDI technology spillover. One view was that FDI technology spillover was an increasing function of technology gap between domestic and foreign-funded enterprises because the larger technology gap would lead to more “catch-up” space for local enterprises (Findlay 1978; Chuang and Hsu 2004; Sjöholm 2007; Lai et al. 2009; Tian et al. 2010; Jordaan 2013; Yin and Zhou 2014). The other view indicated that FDI technology spillover was a decreasing function of the technology gap between domestic and foreign-funded enterprises. The smaller the gap was, the larger the effect gained. The domestic enterprises did not have sufficient absorptive capacity to gain the technology spillover from the foreign-funded enterprises if the gap was excessively large. Furthermore, the domestic enterprises could not use much advanced technology because of their backward technology and lower emulating ability (Lapan and Bardhan 1973; Kokko et al. 1996; Blalock and Gertler 2009; Sawada 2010; Zhang 2013; Hu et al. 2013). Although many scholars recently probed into FDI technology spillover and the threshold effect of technology gap in the country level, the question of FDI technology spillover and the threshold effect of the technology gap in the regional and industrial sector level deserve to be studied further, particularly in different regional Chinese industrial sectors. So, our research did not make a big contribution of theoretical and methodological study to the literature, but in the empirical study, we firstly divided the Chinese industrial sector into eastern, central and western regions. Therefore, this paper uses the provincial panel data of the Chinese industrial sector from 2000 to 2011 to study this question.

## Model and data

### Framework and model

Among the literature regarding FDI technology spillover, the most commonly used model was based on the Cobb–Douglas production function, which estimated the contribution of FDI to the output. Therefore, this paper also acts in accordance with this model; the model is:

1 $$Y_{it}^{D} = A_{it}^{D} (K_{it}^{D} )^{\alpha } (L_{it}^{D} )^{\beta }$$

Additionally, supposing the technology level $A_{it}^{D}$ can be decided by two aspects, its technical progress and the FDI technology spillover, therefore, the model is:

2 $$A_{it}^{D} = e^{c} (R_{it}^{D} )^{\gamma } (FDI_{it} )^{\theta }$$

Then, taking Eq. (2) into Eq. (1) and taking the logarithm on both sides of the equation, the empirical model is:

3 $$LnY_{it}^{D} = C + \alpha LnK_{it}^{D} + \beta LnL_{it}^{D} + \gamma LnR_{it}^{D} + \theta LnFDI_{it} + \varepsilon$$

$Y_{it}^{D}$, $K_{it}^{D}$, $L_{it}^{D}$ and $R_{it}^{D}$ represent the industrial added value of the domestic enterprises, the annual average net value of the fixed assets of the domestic enterprises, the average annual number of employees of the domestic enterprises and the research and development (R&D) funds of the domestic enterprises, respectively, where superscript *D* denotes the domestic enterprises (differing from the foreign one, *F*), and the subscripts *i* and *t* represent the province and year, respectively. Furthermore, the positive coefficient *θ* indicates that the FDI has had positive spillover on the domestic industrial sector and vice versa.

As noted above, the technology gap is the threshold variable in this paper. By reviewing the related literature, there are several methods to measure the technology gap. For example, Zhang (2008) and Guo (2013) use the ratio of the capital density per unit labor of foreign enterprises to domestic enterprises, Jabbour and Mucchielli (2007) use the difference between the average total factor productivity (TFP) and the domestic enterprises TFP, and Tian and Lu (2014) use the ratio of the GDP per unit labor of foreign enterprises to domestic enterprises. All of these three methods have own advantage on measuring the technology gap, but the FDI technology spillover is estimated by the contribution of FDI to the output, so the measurement of technology gap should bring into correspondence with the measurement of the spillover at some aspects. So, this paper acts in accordance with the third method and chooses the ratio of the industrial value-added per capital of foreign enterprises to the domestic enterprises as the indicator of technology gap; therefore, the variable of *TGap* in the empirical model is:

4 $$TGap_{it} = (Y_{it}^{F} /L_{it}^{F} )/(Y_{it}^{D} /L_{it}^{D} )$$

To determine the impact that the threshold effect of the technology gap exerts upon FDI technology spillover, we adopt the threshold regression model of Hansen (1999, 2000) and replace the term of $LnFDI_{it}$ with the interaction term $I = LnFDI_{it} \cdot TGap_{it}$ in Eq. (3). Then, our main estimating model is:

5 $$LnY_{it}^{D} = C + \alpha LnK_{it}^{D} + \beta LnL_{it}^{D} + \gamma LnR_{it}^{D} + \eta \cdot I + \varepsilon$$

The FDI technology spillover is reflected by the partial effect of the variable FDI on the domestic industrial enterprises. Then, we conduct empirical estimation and research whether the threshold effect of the technology gap exists in the FDI technology spillover process in different regional Chinese industrial sectors.

In this paper, the threshold effect indicates that the FDI technology spillover process is different under different technology gap levels. For example, when the technology gap surpasses a critical value, the sign or the magnitude of the coefficient significantly changes. Whether we adopt the multiple threshold model or a single threshold model depends on the number of the threshold values.

This paper tests one, two and three thresholds and selects a double-threshold model at last, which will be illustrated in detail in next part, to estimate the threshold effect. The double-threshold model is:

6 $$\begin{aligned} LnY_{it}^{D} & = C + \alpha LnK_{it}^{D} + \beta LnL_{it}^{D} + \gamma LnR_{it}^{D} + \eta_{1} \cdot I\left( {TGap_{it} \le \lambda_{1} } \right) \\ & \quad + \eta_{2} \cdot I\left( {\lambda_{1} < TGap_{it} < \lambda_{2} } \right) + \eta_{3} \cdot I\left( {TGap_{it} \ge \lambda_{2} } \right) + \varepsilon \\ \end{aligned}$$

In the model, $\lambda_{1}$ and $\lambda_{2}$ represent the two threshold values.

### Model estimation and testing method

When we estimate the model with all possible values of the threshold variable (*TGap*), the estimator for the threshold value should correspond to that yielding the smallest sum of squared errors (SSE). As for the double-threshold model, we should take the following three-stage regressions and obtain the two threshold values.

In the first stage, we find the estimator of the threshold effect through the single-threshold model, so we assume that the model is:

7 $$LnY_{it}^{D} = C + \alpha LnK_{it}^{D} + \beta LnL_{it}^{D} + \gamma LnR_{it}^{D} + \eta^{\prime}_{1} \cdot I\left( {TGap_{it} \le \lambda_{1} } \right) + \eta^{\prime}_{2} \cdot I\left( {TGap_{it} > \lambda_{1} } \right) + \varepsilon$$

and the estimator of the threshold should correspond to the value $\lambda^{ * }$ yields the smallest SSE. Let $S_{n} (\lambda )$ represent the SEE.

In the second stage, assuming that the estimator $\lambda^{ * }$ is equal to $\hat{\lambda }_{1}$ in the double-threshold model, so the second estimator with the criterion:

8 $$\hat{\lambda }_{2} = \arg \min S_{n} (\lambda^{ * } ,\lambda_{2} )$$

In the third stage, the $\hat{\lambda }_{1}$ could be improved to be a refinement estimator. By taking the second-stage estimator $\hat{\lambda }_{2}$, we can obtain the following refinement estimator of $\hat{\lambda }_{1}$:

9 $$\mathop {\lambda_{1} }\limits^{ \wedge } = \arg \min S_{n} (\lambda_{1} ,\hat{\lambda }_{2} )$$

The two threshold values can be obtained after the above three-stage regressions. Subsequently, it is necessary to test whether the threshold effects are statistically significant so that the number of threshold can be determined.

Firstly, the null hypothesis of no threshold effect can be represented by the linear constraint $H_{0} :\eta_{1}^{{\prime }} = \eta_{2}^{{\prime }}$. Under *H*_0_, we can obtain the SEE (S_0_) by estimating Eq. (7), and the actual Lagrange Multiplier test statistic of *H*_0_ is:

10 $$F_{1} = (S_{0} - S_{n} (\hat{\lambda }_{1} ))/\hat{\sigma }^{2}$$

Among the Eq. (10), the $S_{n} (\hat{\lambda }_{1} )$ is the minimized SEE under the single threshold assumption, and the $\hat{\sigma }^{2}$ is the corresponding variance estimator of the residual. Under *H*_0_, the threshold $\hat{\lambda }_{1}$ is not identified, therefore the classic tests have non-standard distributions and the critical level can not be gained from the standard $\chi^{2}$ distribution tables. But we can follow Hansen (1999)’s method and use a bootstrap procedure to compute the stimulated LM statistic so that we can gain the *P* value. The null hypothesis of no threshold effect is rejected if the P value is small than the desired critical value.

Then, we take the null hypothesis of only one threshold based on the Eq. (6), the $H_{0}^{{\prime }} :\eta_{2} = \eta_{3}$, to test the discrimination between one and two threshold. Under *H*′_0_, we can obtain the SEE ($S_{1} (\hat{\lambda }_{1} )$) by estimating Eq. (6), and the actual Lagrange Multiplier test statistic of *H*′_0_ is:

11 $$F_{2} = (S_{1} (\hat{\lambda }_{1} ) - S_{n} (\hat{\lambda }_{1} ,\hat{\lambda }_{2} ))/\hat{\sigma }^{{{\prime }2}}$$

$S_{n} (\hat{\lambda }_{1} ,\hat{\lambda }_{2} ))$ is the minimized SEE for Eq. (7), and the $\hat{\sigma }^{{{\prime }2}}$ is the corresponding variance estimator of the residual. Similarly, following Hansen (1999), we use the bootstrap technique to gain the P value. The hypothesis of one threshold is rejected in favor of two thresholds if *F*_2_ is sufficiently large. The test for M + 1 thresholds can be continued if the null hypothesis of M thresholds cannot be rejected using the same logic. Finally, we can confirm the number of thresholds.

### Variables and data

This paper chooses the industrial panel data of 30 provinces (Tibet’s data are not included) from 2000 to 2011. The original data originates from the “China Statistical Yearbook”, the “China Industry Economy Statistical Yearbook”, the “China Statistical Yearbook on Science and Technology” and the National Database.

The variables in this paper are as follows: Output (Y, 0.1 Billion Yuan) is measured by the industrial value-added; Capital (K, 0.1 Billion Yuan) is a stock variable and is measured by the annual average net value of fixed assets; Labor (L, 10 thousand employees) is measured by the average annual number of employees. The values of the domestic enterprise variables ($Y_{it}^{D}$, $K_{it}^{D}$, $L_{it}^{D}$) are calculated by subtracting the values of the three types of foreign-invested enterprises ($Y_{it}^{F}$, $K_{it}^{F}$, $L_{it}^{F}$) from the values of all state-owned and non-state-owned enterprises above the designated size industrial enterprises. This paper adopts the sum of foreign capital and Hong Kong, Macao and Taiwan regions’ capital to measure the main variable of foreign direct investment (FDI, 0.1 Billion Yuan); R&D (R&D, 0.1 Billion Yuan) is measured by the R&D funds of domestic enterprises. The original data above are determined by the current price for each year. To eliminate the price impact and obtain the real values, output is deflated by the ex-factory price index of industrial goods; furthermore, capital, FDI and R&D are deflated by the price index of investment in fixed assets.

In addition, according to the economic development level and the speed of economic development, the National Bureau of Statistics divides the 30 provinces into eastern, central and western regions. The eastern region includes Beijing, Tianjin, Hebei, Liaoning, Shanghai, Jiangsu, Zhejiang, Fujian, Shandong, Guangdong and Hainan; the central region includes Shanxi, Jilin, Heilongjiang, Anhui, Jiangxi, Henan, Hubei and Hunan; the western region includes Inner Mongolia, Guangxi, Chongqing, Sichuan, Guizhou, Yunnan, Shaanxi, Gansu, Qinghai, Ningxia and Xinjiang. Therefore, in accordance with this division method, this paper divides the Chinese industrial sector into eastern, central and western industrial sectors. The statistical description of the main variables are shown in Table 1.

**Table 1:** The statistical description of the main variables

Variable	Mean	Std. dev.	Min	Max	Observations
Y					
Eastern region	3252.21	3358.72	53.20	17,636.50	132
Central region	1905.77	1659.78	246.94	9274.77	96
Western region	1003.86	1215.41	62.74	7768.45	132
K					
Eastern region	3533.47	2662.09	154.20	14,530.20	132
Central region	2552.01	1503.35	704.03	8923.31	96
Western region	1506.93	1102.68	224.41	6338.23	132
L					
Eastern region	271.62	210.28	8.59	765.75	132
Central region	185.26	83.97	87.21	501.08	96
Western region	85.24	60.72	12.63	346.79	132
FDI					
Eastern region	1374.08	1651.90	17.08	7165.42	132
Central region	147.85	115.87	10.20	497.14	96
Western region	58.54	65.83	0.63	296.36	132
R&D					
Eastern region	84.66	91.18	0.55	432.75	132
Central region	33.33	28.30	2.27	136.17	96
Western region	14.31	15.61	0.25	70.15	132
TGap					
Eastern region	1.25	0.43	0.66	2.75	132
Central region	1.74	0.96	0.53	5.23	96
Western region	1.48	0.95	0.41	7.27	132

## Empirical analysis

### Test and estimation threshold values

To ensure the number of thresholds, we used the software of STATA 12.0 and Eq. (5) was estimated by least squares, allowing for zero, one, two, and three thresholds of the eastern, central and western domestic industrial sector. The LM test statistics, the F value, and their bootstrap P values are shown in Table 2. We find that for the test of the eastern domestic industrial sector for the null threshold, the F value is 11.144 and the critical value at the 5 % significance level is 10.059. The F value is larger than the critical value; therefore, it is significantly rejected, with a bootstrap P value of 0.037. Furthermore, the test for the sole single threshold, the F value, is also significantly rejected, with a bootstrap P value of 0.057 at the 10 % significance level. However, for the LM test statistic for the double threshold, the F value is not statistically significant, with a bootstrap P value of 0.733. Therefore, we can conclude that there is strong evidence that the double threshold effects of the technology gap exist in the FDI technology spillover process to the eastern domestic industrial sector. We also perform the same test for the central and western domestic industrial sectors. Additionally, we find that two thresholds in both the central and western domestic industrial sectors also exist.

**Table 2:** The results of the test for threshold effects

Threshold effect	F value	P value	Critical value		
			1 %	5 %	10 %
Eastern region					
Single threshold	11.144**	0.037	17.089	10.059	6.775
Double threshold	16.210*	0.057	44.210	18.906	12.017
Triple threshold	−0.000	0.733	0.000	0.000	0.000
Central region					
Single threshold	13.800**	0.013	14.884	8.025	6.232
Double threshold	57.552***	0.000	15.150	5.075	0.752
Triple threshold	0.000	0.313	0.000	0.000	0.000
Western region					
Single threshold	21.920*	0.057	30.285	23.509	18.305
Double threshold	17.856*	0.073	37.893	20.581	15.877
Triple threshold	0.000	0.243	0.000	0.000	0.000

The stars *, **, and *** indicate statistical significance at the 10, 5 and 1 % levels, respectively

Then, we use the industrial panel data of 30 provinces from 2000 and 2011 and adopt the above double-threshold regression model to estimate the critical threshold values of the eastern, central and western domestic industrial sector. Table 3 reports the estimation results.

**Table 3:** The estimations of threshold value and confidence interval

Threshold	Estimate	95 % confidence interval
Eastern		
λ1	1.254	[1.061, 2.163]
λ2	2.163	[0.820, 2.502]
Central		
λ1	1.516	[1.336, 1.866]
λ2	2.694	[1.365, 2.694]
Western		
λ1	1.635	[0.642, 1.758]
λ2	2.714	[2.670, 3.594]

According to the two threshold values, the three regions are divided into three groups. Table 4 reports the number of provinces of each year that fall into the three groups in different regions. From Table 4, we can observe that the number of samples below the first threshold value increases gradually each year in each region; at the same time, the number of samples above the second threshold value decreases gradually each year in each regional industrial sector. This finding means that the technology gap between the domestic enterprises and the foreign-funded enterprise decreases gradually each year in each regional industrial sector.

**Table 4:** Number of different region samples in each group by year

	Eastern region			Central region			Western region		
	TGap < λ1	λ1 < TGap < λ2	TGap > λ2	TGap < λ1	λ1 < TGap < λ2	TGap > λ2	TGap < λ1	λ1 < TGap < λ2	TGap > λ2
2000	4	4	3	1	5	2	5	2	4
2001	6	3	2	1	6	1	3	6	2
2002	4	6	1	2	5	1	3	6	2
2003	5	5	1	2	5	1	3	6	2
2004	5	6	0	4	3	1	7	3	1
2005	8	3	0	5	2	1	9	2	0
2006	8	3	0	6	1	1	9	2	0
2007	8	2	1	6	1	1	10	1	0
2008	9	1	1	6	1	1	10	1	0
2009	8	3	0	6	1	1	10	1	0
2010	8	3	0	7	0	1	10	1	0
2011	8	3	0	7	0	1	10	1	0

### The regression results of FDI technology spillover

After confirming the existence of two threshold values, we must research the relation between the threshold effect of the technology gap and FDI technology spillover; finally, to determine the economic meaning of those threshold values, we must compare the different impacts of the technology gap on technology spillover in the three sub-groups. As shown in Table 5, the results are as follows:

**Table 5:** The estimates of FDI technology spillovers for the three regions 2000–2011

ln Y	East	Middle	West
ln K	0.945***	0.511***	0.73***
	0.1	0.12	0.06
ln L	−0.042	0.435***	0.036
	0.06	0.10	0.06
ln R&D	0.087**	0.298***	0.257***
	0.05	0.07	0.04
I(TGap ≤ λ1)	0.150***	0.163***	0.124***
	0.03	0.05	0.04
I(λ1 ≤ TGap ≤ λ2)	0.117***	0.113**	0.075
	0.03	0.05	0.05
I(TGap ≥ λ2)	0.086**	0.222***	0.001
	0.04	0.05	0.05
C	−0.861	−0.593	0.212
	0.47	0.42	0.36
Adjusted R square	0.9623	0.9444	0.9549
F-statistics	514.07	408.00	357.62
(Pr > F)	0.000	0.000	0.000
F test for no fixed effects	3.44	9.85	4.45
(Pr > F)	0.0106	0.000	0.0004
N	132	96	132

The stars ** and *** indicate statistical significance at the 5 % and 1 % levels, respectively

From Table 5, we can observe that the coefficients of the interaction item are nearly positive, namely the FDI technology spillover to the domestic industrial sector is positive. For the eastern domestic industrial sector, the technology spillover is significant in each technology gap level; when the technology gap is less than 1.254, the technology spillover is 0.150. When the technology gap is between 1.254 and 2.163, the technology spillover decreases to 0.117. When the technology gap exceeds 2.163, the technology spillover decreases to 0.086. Regarding the central domestic industrial sector, the technology spillover is also significant at each technology gap level. When the technology gap is less than 1.516, the technology spillover is 0.163. When the technology gap is between 1.516 and 2.694, the technology spillover decreases to 0.113. When the technology gap is over 2.694, the technology spillover rises to 0.222. Regarding the western domestic industrial sector, when the technology gap is less than 1.635, the technology spillover is 0.124. When the technology gap is between 1.635 and 2.714, the technology spillover is not significant and decreases to 0.075. When the technology gap is over 2.714, the technology spillover is also not significant and decreases to 0.001.

Regarding the other variables, the estimated coefficient in each region regression model is nearly consistent, which indicates that capital, labor, and R&D have a relatively stable influence on the domestic industry added value. These factors all have positive and significant effects on the domestic industry added value. However, the influence of labor on the eastern domestic enterprises is negative but not significant. Because the investment of the eastern industrial sector that focuses on the development of capital intensive industries and the absorption of the labor force is very limited and excludes labor, the non-absorbed labor force is unemployed, and unemployment will hinder the improvement of the eastern industrial sector and produce negative effects on the domestic industry added value.

**Fig. 1:**
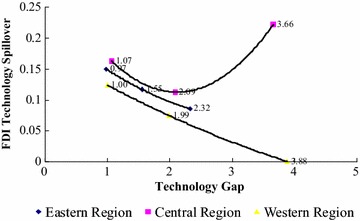
The relation between FDI technology spillover and technology gap in different regional industrial sectors. The *line of yellow points* represents the relation between FDI technology spillover and technology gap in western region; the *line of blue points* represents the relation between FDI technology spillover and technology gap in eastern region; the *curve line of pink points* represents the relation between FDI technology spillover and technology gap in central region

To reflect the relation between FDI technology spillover and the technology gap directly and distinctly, this paper takes the technology spillover of FDI and technology gap as the ordinate and the abscissa, respectively. Figure 1 shows the relation.

From Fig. 1, we can observe that the technology gap of the eastern region is smaller, between 0.97 and 2.32 generally; however, the central and western regions are larger, between 1.07 to 3.66 and between 1.00 to 3.88, respectively. In addition, with the technology gap increasing, the technology spillover is decreasing in the eastern and western domestic industrial sectors. When the technology gap approaches 3.88, the FDI technology spillover in the western domestic industrial sectors nears zero. We can call the FDI technology spillover a decreasing function of the technology gap. Furthermore, we find that, with the technology gap increasing, the technology spillover is decreasing; however, when the technology gap exceeds 2.09 approximately, the technology spillover is increasing gradually in the central domestic industrial sectors. We can state that the FDI technology spillover is a concave curve function of the technology gap.

Moreover, we have observed that the technology gap is decreasing gradually each year in each regional industrial sector from Table 4. Combining Fig. 1, we can also find that the FDI technology spillover has been increasing gradually in each domestic industrial sector in recent years.

## Conclusions and policy recommendations

In this paper, we propose a double-threshold model and inspect the threshold effect of FDI technology spillover by using the industrial panel data of 30 Chinese provinces from 2000 to 2011. The empirical results support that there is a threshold effect in terms of the technology gap that exists in the FDI technology spillover process in different regional Chinese industrial sectors, and there are two technology gap threshold values that exist in the domestic industrial sectors of each region. Therefore, there are two main conclusions that can be derived. One conclusion is that the FDI technology spillover is a decreasing function of THE technology gap in both the eastern and western domestic industrial sector and is a concave curve function of the technology gap in the central domestic industrial sector. Another conclusion is that the FDI technology spillover has been increasing gradually in each domestic industrial sector in recent years.

Therefore, there are four policy implications that can be recommended.

First, considering that the FDI technology spillover is positive and significant in each regional industrial sector, we should introduce FDI activity.

Second, the threshold effect of the technology gap exists in the FDI technology spillover process. In addition to the increasing technology gap, technology spillover decreases gradually; particularly in the western industrial sector, the technology spillover will reduce and tend to zero. Therefore, the industrial sector should introduce foreign-invested enterprises whose technology level is slightly higher than domestic enterprises.

Third, the central industrial sector should introduce the foreign-invested enterprises whose technology level is slightly higher than the domestic enterprises and the eastern and western industrial sector. However, it is also necessary to introduce those enterprises with higher technology that can help domestic enterprises to exert their own imitation and learning effects to improve their technology levels. Furthermore, this behavior can also increase the technology spillover.

Finally, the regional domestic industrial sector should put much more capital and R&D in its production and improve technology; this can heavily promote the industrial added value growth.

## References

[CR1] Blalock G, Gertler PJ (2009). How firm capabilities affect who benefits from foreign technology. J Dev Econ.

[CR01] Blomström M (1986). Foreign investment and productive efficiency: the case of mexico. J Ind Econ.

[CR2] Caves RE (1974). Multinational firms, competition, and productivity in host-country markets. Economica.

[CR3] Chuang YC, Hsu PF (2004). FDI, trade, and spillover efficiency: evidence from China’s manufacturing sector. Appl Econ.

[CR4] Cohen WM, Levinthal DA (1989). Innovation and learning: the two faces of R&D. Soc Sci Electron Publ.

[CR05] Dimelis S, Louri H (2002). Foreign ownership and production efficiency: a quantile regression analysis. Oxf Econ Pap.

[CR5] Findlay R (1978). Relative backwardness, direct foreign investment, and the transfer of technology: a simple dynamic model. Q J Econ.

[CR04] Girma S (2005). Absorptive capacity and productivity spillovers from fdi: a threshold regression analysis. Oxf Bull Econ Stat.

[CR03] Globerman S (1979). Foreign direct investment and spillover efficiency benefits in canadian manufacturing industries. Can J Econ/revue Canadienne D`economique.

[CR6] Guo QR (2013). Regional difference of the technology spillover from FDI and the absorptive capacity—a perspective based on the threshold panel data model. J Ind Technol Econ.

[CR7] Hansen EB (1999). Threshold effect in non-dynamic panels: estimation, testing, and inference. J Econom.

[CR8] Hansen EB (2000). Sample splitting and threshold estimation. Econometrica.

[CR9] He J, Xu LD (1999). An empirical research of foreign direct investment spillover effect on Chinese industrial sector. World Econ Pap.

[CR10] Hu ZY, Tang LW, Chen J (2013). Nonlinear effect between FDI spillover and regional technological progress. J Hunan Univ.

[CR11] Jabbour L, Mucchielli JL (2007). Technology transfer through vertical linkages: the case of the Spanish manufacturing industry. J Appl Econ.

[CR02] Javorcik BS, Spatareanu M (2008). To share or not to share: does local participation matter for spillovers from foreign direct investment?. J Dev Econ.

[CR12] Jordaan JA (2013). Firm heterogeneity and technology transfers to local suppliers: disentangling the effects of foreign ownership, technology gap and absorptive capacity. J Int Trade Econ Dev.

[CR13] Jude C (2015) Technology spillovers from FDI. Evidence on the intensity of different spillover channels. World Econ

[CR14] Kokko A (1994). Technology, market characteristics, and spillovers. J Dev Econ.

[CR15] Kokko A, Tansini R, Zejan M (1996). Productivity spillovers from FDI in the Uruguayan manufacturing sector. J Dev Stud.

[CR16] Kuo CC, Yang CH (2008). Knowledge capital and spillover on regional economic growth: evidence from china. China Econ Rev.

[CR17] Lai M, Wang H, Zhu S (2009). Double-edged effects of the technology gap and technology spillovers: evidence from the Chinese industrial sector. China Econ Rev.

[CR18] Lapan H, Bardhan P (1973). Localized technical progress and transfer of technology and economic development. J Econ Theory.

[CR19] Pan W (2003) The spill-over effects of FDI on China’s industrial sectors: a panel data analysis. World Econ

[CR21] Sawada N (2010). Technology gap matters on spillover. Rev Dev Econ.

[CR22] Sjöholm F (2007). Technology gap, competition and spillovers from direct foreign investment: evidence from establishment data. J Dev Stud.

[CR20] Suyanto, Salim R (2013). Foreign direct investment spillovers and technical efficiency in the Indonesian pharmaceutical sector: firm level evidence. Appl Econ.

[CR23] Tian ZY, Lu T (2014) The threshold effect between technology gaps based on the heterogeneity. In: 2014 IEEE international conference on IEEE management of innovation and technology (ICMIT). IEEE, pp 339–344

[CR24] Tian ZY, Jiang KS, Jiang H (2010). The spillover effect of FDI on private enterprises—an empirical study based on interprovincial panel data. R&D Manag.

[CR25] World Investment Report (2015) U.N. conference on trade and development. http://unctad.org/en/Pages/Home.aspx. Accessed 20 Sep 2015

[CR26] Xie JG (2006). Technical spillovers of foreign direct investment in China: a study based on provinces panel data. China Econ Q.

[CR27] Yi J, Chen Y, Wang C, Kafouros M (2015). Spillover effects of foreign direct investment: how do region-specific institutions matter?. Manag Int Rev.

[CR28] Yin JH, Zhou XY (2014). Empirical research on reverse technology spillover effect in China’s outward FDI—from the perspective of technology gap threshold. Sci Res Manag.

[CR29] Yu CL (2011). Intellectual property rights protection and international R&D spillovers. World Econ Study.

[CR30] Zhang Y (2008). The local difference of FDI technology spillovers and the threshold characters of absorbing capacity. J Quant Tech Econ.

[CR31] Zhang WA (2013). Technology gap and FDI spillover effects: an empirical study of China’s industrial sector. J Yunnan Univ Finance Econ.

[CR32] Zhang C, Guo B, Wang J (2014). The different impacts of home countries characteristics in FDI on Chinese spillover effects: based on one-stage SFA. Econ Model.

